# Racial Disparity in Anthracycline-induced Cardiotoxicity in Breast Cancer Patients

**DOI:** 10.3390/biomedicines11082286

**Published:** 2023-08-17

**Authors:** Swetha Balaji, Antu K. Antony, Harry Tonchev, Giorgia Scichilone, Mohammed Morsy, Hania Deen, Imaduddin Mirza, Mohamed M. Ali, Abeer M. Mahmoud

**Affiliations:** 1 Division of Endocrinology, Diabetes, and Metabolism, Department of Medicine, College of Medicine, University of Illinois at Chicago, Chicago, IL 60612, USA; sbalaji2@uic.edu (S.B.); antuk@uic.edu (A.K.A.); harrytonchev@yahoo.com (H.T.); gscich2@uic.edu (G.S.); mmorsy3@uic.edu (M.M.); hdeen2@uic.edu (H.D.); mmirza24@uic.edu (I.M.); mali37@uic.edu (M.M.A.); 2 Department of Kinesiology, College of Applied Health Sciences, University of Illinois at Chicago, Chicago, IL 60612, USA

**Keywords:** cardiotoxicity, anthracyclines, breast cancer, genetic polymorphism, racial disparity, socioeconomic determinants, cardiometabolic health

## Abstract

Breast cancer has become the most common cancer in the US and worldwide. While advances in early detection and treatment have resulted in a 40% reduction in breast cancer mortality, this reduction has not been achieved uniformly among racial groups. A large percentage of non-metastatic breast cancer mortality is related to the cardiovascular effects of breast cancer therapies. These effects appear to be more prevalent among patients from historically marginalized racial/ethnic backgrounds, such as African American and Hispanic individuals. Anthracyclines, particularly doxorubicin and daunorubicin, are the first-line treatments for breast cancer patients. However, their use is limited by their dose-dependent and cumulative cardiotoxicity, manifested by cardiomyopathy, ischemic heart disease, arrhythmias, hypertension, thromboembolic disorders, and heart failure. Cardiotoxicity risk factors, such as genetic predisposition and preexisting obesity, diabetes, hypertension, and heart diseases, are more prevalent in racial/ethnic minorities and undoubtedly contribute to the risk. Yet, beyond these risk factors, racial/ethnic minorities also face unique challenges that contribute to disparities in the emerging field of cardio-oncology, including socioeconomic factors, food insecurity, and the inability to access healthcare providers, among others. The current review will address genetic, clinical, and social determinants that potentially contribute to this disparity.

## 1. Introduction

Breast cancer is becoming more common and pervasive in the United States, with incidence rates increasing by 0.5% yearly [1]. It ranks second among cancer-related mortality in women, particularly among African American women, who have a higher risk of acquiring breast cancer before age 40 and a higher mortality rate [1]. Although early detection and treatment have reduced mortality, the number of African Americans developing aggressive types of breast cancer continues to rise [2]. The presence or absence of molecular markers for hormone receptors (estrogen and progesterone receptors) and human epidermal growth factor 2 (Her2-neu) differentiates breast cancer subtypes [3]. These molecular subtypes, along with characteristics like the cancer stage, underlying genetic factors, and responsiveness to neoadjuvant therapy before surgery, influence treatment decisions [4].

The most efficient and often used chemotherapy agents for treating breast cancer are those in the anthracycline class [5]. Despite their widespread usage in treating breast cancer, anthracyclines have numerous side effects, on top of which is cardiotoxicity [6]. Anthracycline-induced cardiotoxicity can be either acute or chronic. While acute cardiotoxicity is typically infrequent and dose-independent, chronic anthracycline-induced cardiotoxicity is a more frequent and dose-dependent outcome that eventually promotes the development of heart failure [7]. At the molecular level, anthracyclines promote cardiac muscle damage and endothelial dysfunction by increasing oxidative and nitrosative stress in conjunction with topoisomerase 2-beta inactivation and other molecular alterations discussed in the corresponding section [8]. 

Despite increased breast cancer survival, cardiovascular disease is becoming more common among survivors [9]. Within the population of breast cancer patients who are 50 and older, cardiovascular disease constitutes 35% of non-cancer-related mortality [9,10]. The available data derived from the Surveillance, Epidemiology, and End Results (SEER) program, together with other epidemiological studies, reveal that individuals who have survived breast cancer exhibit a higher prevalence of cardiovascular disease compared to women who have not been diagnosed with breast cancer [9,11]. Additionally, breast cancer survivors had a greater mortality risk from cardiovascular disease than those without cancer [12]. The contribution of anthracyclines to the development and exacerbation of cardiovascular diseases is highlighted in the most recent European Society of Cardiology (ESC) guidelines on cardio-oncology (2022) [13]. Anthracycline is identified as one of the cancer treatments most likely to induce a significant decrease in left ventricular (LV) ejection fraction (LVEF), cardiomyopathy, and cardiac ischemia, ultimately leading to heart failure. Citing over 40 genetic variants associated with anthracycline-induced cardiac toxicity, the ESC guidelines emphasize the significance of taking into consideration numerous variables that can influence the outcome of anthracycline administration. These guidelines also refer to the underrepresentation of minority populations in ongoing clinical trials and emphasize the need to conduct bigger anthracycline-centered trials that include high-risk populations. Moreover, these guidelines are intended to assist oncologists in making informed decisions regarding treatment doses and regimens, preventing the premature cessation of therapy for patients who may benefit from prolonged therapy, and preventing severe complications in patients who are at a greater risk of developing cardiac toxicity. In a nutshell, the ESC guidelines encourage classifying patients based on several factors that determine their risk of anthracycline-induced cardiac toxicity, which is expected to facilitate the early implementation of personalized preventive strategies. In line with these recommendations, we deemed it necessary to discuss the biological and non-biological factors that may explain the racial/ethnic disparities in anthracycline-induced cardiotoxicity and may inform future decisions regarding minority patients.

Preexisting cardiac conditions and established cardiovascular risk factors influence the risk of anthracycline-induced cardiotoxicity [14]. African American and Hispanic women are believed to have a higher cardiovascular risk than their White counterparts. A study by Zhang et al. [15] showed a greater incidence of heart failure in breast cancer survivors of African American (12%) and Hispanic heritage (11%) compared to White patients (6%). This situation can be attributed to genetic, environmental, and socioeconomic disparities that exacerbate and contribute to the discrepancies in risk factors [16]. Thus, breast cancer patients from ethnic minorities face an increased risk of anthracycline-induced cardiotoxicity.

Aside from biological risk factors, socioeconomic, environmental, behavioral, and structural disparities all impact overall mortality and morbidity in breast cancer survivors [17]. These factors have been demonstrated to have a more significant impact on racial/ethnic minority groups. Social determinants of health affect not only the incidence of breast cancer but also its progression and survival outcomes, as well as the response to therapy [18]. It is critical to fully comprehend the impact of these factors on the observed discrepancy in cardiotoxicity in response to anthracycline in breast cancer patients. To this purpose, the representation of diverse ethnic minority groups is required to comprehend numerous contributing elements. This review article discusses various aspects that may contribute to racial disparities in anthracycline-induced cardiotoxicity among African Americans and other ethnic minority groups.

## 2. Clinical Manifestations of Anthracycline-Induced Cardiotoxicity

The efficacy of anthracyclines in treating breast cancer is hampered by the cumulative dose-dependent cardiotoxicity that they commonly cause, eventually resulting in heart failure. For instance, doxorubicin, the prototypical anthracycline chemotherapeutic agent, causes a dose-dependent reduction in left ventricular ejection fraction (LVEF) when the cumulative dose exceeds 350 mg/m^2^ [7]. Other studies showed that doxorubicin reduces the LVEF by around 6 to 33% when the dose is 250 to 400 mg/m^2^ [19,20]. The pathogenesis of anthracycline-induced toxicity includes cardiomyopathy, arrhythmias, pericardial effusion, heart failure, and sudden death [21]. In breast cancer patients receiving anthracyclines, the three-year mortality rate due to cardiac arrest or heart failure was 3%, compared to 1% in an age-matched cohort that did not receive chemotherapy [22].

Factors that increase the cardiotoxicity risk in patients receiving anthracyclines include older age (>60 years), baseline LVEF < 55%, combining chemotherapy and radiotherapy, sequential use of Her-2 antagonists, and preexisting cardiac diseases [23]. However, even without these risk factors, women from ethnic minorities have higher rates of cardiotoxicity in response to anthracyclines [24], necessitating further research into the biological and non-biological factors that might be responsible for this disparity. In a study by Al-Sadawy et al. [25], the risk of cardiotoxicity, as defined by clinical heart failure or an asymptomatic reduction in LVEF, was substantially higher in African American breast cancer patients than in White patients (OR, 2.10; 95% CI, 1.42–3.10). Even after adjusting for cardiovascular risk factors, income, level of education, and insurance status, this association remained significant. Similarly, in a follow-up study by Hasan et al. [26], African American breast cancer survivors had a higher incidence of cardiotoxicity, which was defined as the development of congestive heart failure or LVEF ≤ 45, compared to White patients. Also, African American breast cancer survivors exhibited higher all-cause mortality compared with White patients; 30% of this increase was explained by preexisting hypertension in the former group [27]. Troeschel et al. [28] observed that African American women exhibited a higher projected 20-year cumulative incidence of mortality linked to cardiovascular disease following anthracyclines in comparison to White patients. Comparable discrepancies have been documented within the Hispanic/Latina demographic, which represents the second most populous racial/ethnic group in the United States. In their study, Hu et al. [29] demonstrated that Hispanic breast cancer survivors exhibited a higher risk of cardiovascular illnesses (HR = 1.94) compared to non-Hispanic White breast cancer survivors (HR = 1.38) within a timeframe of 1–5 years following their cancer diagnosis.

While cardiomyocytes have been shown to be the primary target of anthracycline-mediated toxicity, recent studies have demonstrated that anthracyclines also affect cardiac fibroblasts, cardiac progenitor cells, and endothelial cells [14]. Anthracycline-induced cardiomyopathy is characterized by myocellular apoptosis and necrosis, resulting in extensive damage to the left and right ventricles [14,30]. These effects manifest as gradual impairments in both systolic and diastolic functions, progressing to dilated cardiomyopathy and, ultimately, heart failure [31]. The cardiotoxic manifestations of anthracycline treatment were divided into those that occur early in the course of treatment (within the first year) and those that occur later than a year after the treatment’s initiation [32]. Early clinical signs include arrhythmias (ventricular or supraventricular), atrioventricular block, and pericarditis–myocarditis syndrome; they were primarily reported in response to an anthracycline analog, mitoxantrone. These symptoms are thought to be caused by acute myocardial damage after infusion [33,34]. Troponin elevation and mild ventricular dysfunction have also been reported and have been shown to predict the development of ventricular dysfunction at later time points. A prospective study with 2625 patients showed that lower LVEF measured at the end of chemotherapy was an early independent predictor of late-stage cardiotoxicity. Yet, more investigations are required to determine the predictive value of early signs of cardiotoxicity [35,36].

Early cardiotoxicity induced by anthracyclines could develop without clinical symptoms. The cause of this lack of symptoms is primarily due to compensatory mechanisms mediated by ventricular remodeling in an effort to preserve functional capacity. Prolonged compensatory mechanisms, on the other hand, will result in ventricular wall thinning and dilated cardiomyopathy. The onset of these changes coincides with the appearance of clinical symptoms. Unfortunately, by the time late manifestations of cardiotoxicity, such as dyspnea, fatigue, orthopnea, and edema, develop, cardiotoxicity is mostly irreversible, and progression to heart failure is more likely [37]. Therefore, it is essential to frequently monitor cardiotoxicity in patients receiving anthracyclines. Frequent heart evaluation with echocardiography helps detect asymptomatic declines in cardiac functions during the first year of anthracycline treatment [32]. Echocardiography is a feasible, widely available approach, easily reproducible, and free from radiation exposure [19]. One of the critical cardiotoxicity parameters that can be detected with echocardiography is LVEF [22]. The ejection fraction refers to the proportion of blood that is expelled from the heart during each cardiac cycle. A decrease in LVEF is defined as less than 51% in men and 53% in women, with less than 40% indicating uncompensated heart failure or cardiomyopathy in either gender [23]. Because impaired left ventricular systolic function does not demonstrate intrinsic myocardial contractility, LEVF can still be normal [38]. As a result, assessing myocardial strain can overcome the limitations of LVEF in assessing left ventricular systolic function.

Global longitudinal strain (GLS) is the most used type of strain to clearly define left ventricular systolic function [38]. It has well-established diagnostic and prognostic values and can detect early alterations resulting from cardiotoxicity. Anthracycline-induced damage is assessed using two-dimensional speckle-tracking echocardiography (2D STE) with GLS [23]. In addition to systolic function, diastolic function should also be evaluated. Many studies have found abnormalities in the left ventricular diastole before systolic dysfunction. The E/A ratio (which represents the early (E) and late (A) diastolic filling velocities) and the isovolumic relaxation time are commonly used to assess diastolic dysfunction. Nonetheless, there has been little research on early diastolic dysfunction in anthracycline-induced cardiac toxicity [39].

Anthracyclines also impact the right ventricle, the dysfunction of which is an independent prognostic factor in several cardiovascular diseases and a crucial predictor of cardiovascular mortality [40]. However, studies measuring right ventricular dysfunction in response to anthracycline treatment in breast cancer patients are scarce [41]. The lack of research in this area may be attributed to the intricate structure and positioning of the right ventricle, which presents challenges in non-invasively assessing its size and function. The advent of three-dimensional (3DE) and two-dimensional speckle-tracking echocardiography (2DSTE) has facilitated the investigation of right ventricular function. While 3DE is preferable for assessing right ventricular volumes and ejection fractions, 2DSTE is superior for measuring myocardial deformations and is a strong predictor of survival and functional capacity in patients [42]. Laufer-Perl et al. [43] found a substantial decrease (>10%) in the right ventricular GLS and FWLS-PK (free wall longitudinal strain systolic peak) in 40 breast cancer patients at the end of their anthracycline treatment cycle compared to their baseline measurements. In this study, the left ventricular GLS and ejection fraction were normal, indicating that right ventricular damage may precede damage to the left ventricle and confirming the importance of screening for subclinical right ventricular dysfunction [40]. These findings have led to recommendations from the American Society of Clinical Oncology and the British Society of Echocardiography (BSE) that transthoracic echocardiographic assessments of anthracycline-receiving patients be performed every three months during active cancer treatment and once yearly after the active treatment [44].

## 3. Molecular Mechanisms of Anthracycline-Induced Cardiotoxicity

**Figure 1:**
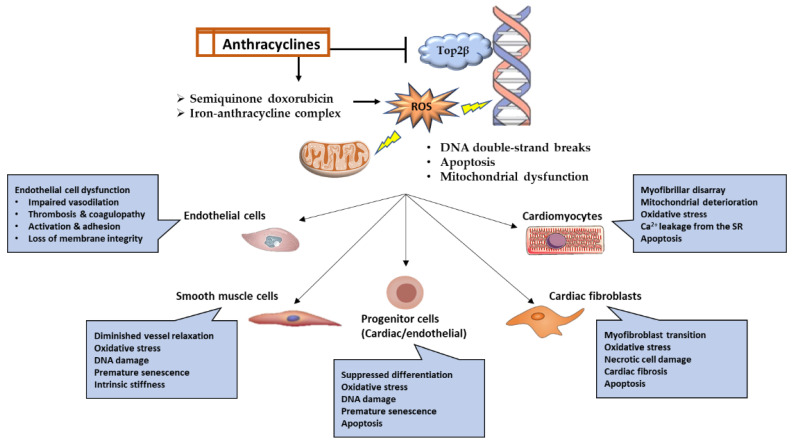
Graphical representation of the molecular pathways and functional outcomes in numerous cell types affected by anthracycline-induced cardiotoxicity.

Anthracyclines have a propensity for mitochondrial accumulation; hence, myocardial tissue is the primary target of anthracyclines due to its high energy demand and, consequently, high mitochondrial density [45]. Although cardiomyocytes are the primary target, research has revealed that anthracyclines can also impact other cell types, including fibroblasts, cardiac progenitor cells, and endothelial cells [14,45]. Anthracyclines produce cardiotoxicity through a wide variety of molecular mechanisms, some of which include the suppression of topoisomerase 2, the production of reactive oxygen species (ROS), alterations in iron metabolism, and changes in Ca^2+^ signaling [45,46]. Myofilament degeneration, myocyte loss, mitochondrial enlargement and fragmentation, and vacuolar degeneration of the sarcoplasmic reticulum are some of the typical pathologies that can be detected in the myocardium in response to anthracyclines [47]. The myocardium of individuals treated with anthracyclines showed evidence of cell death by apoptosis and necrosis, and consequently, serum troponin levels were raised [48]. Since the purpose of this review is not to provide a comprehensive description of these mechanisms, we will only discuss the most prevalent ones (Figure 1), recognizing that new mechanisms are still being discovered and that the precise mechanism underlying anthracycline-induced cardiotoxicity cannot be determined with certainty.

### 3.1. Topoisomerase-2 (Top2)

Anthracyclines intercalate into DNA, forming bulky DNA adducts and crosslinks that interfere with the replication and transcription process [7,49]. Top2 is the main target for doxorubicin, and it has two forms, Top2α, expressed by highly proliferating cells such as cancer cells, and Top2β, expressed by quiescent cells such as cardiomyocytes. Doxorubicin inhibits the catalytic activity of Top2β and stabilizes its intermediary form, causing covalent binding with DNA and persistent DNA double-strand breaks (DSBs). The broken DNA will eventually lead to the recruitment and activation of p53-induced apoptotic cell death. Furthermore, disturbances in the function of Top2β will interfere with the transcription of genes critical for mitochondrial biogenesis, such as peroxisome proliferator-activated receptor gamma receptor co-activators (*PPARδ*), and mitochondrial function, such as NADH: ubiquinone oxidoreductase subunit A3 (*Ndufa3*), succinate dehydrogenase complex flavoprotein subunit A (*Sdha*), and ATP synthase F1 subunit alpha (*Atp5a1*) [7].

### 3.2. Reactive Oxygen Species

The generation of reactive oxygen species (ROS) is another mechanism through which anthracyclines can damage DNA [7,46], leading to base mismatch, point mutations, nucleotide oxidation, and DNA single-stranded breaks. Doxorubicin exhibits a selective tendency to accumulate within mitochondria owing to its high affinity for cardiolipin, a phospholipid mostly present in the inner mitochondrial membrane. Doxorubicin can be converted to the semiquinone form via NADPH oxidase and nitric oxide synthase in the cytoplasm and the electron transport chain in the mitochondria. This semiquinone doxorubicin form is unstable and frequently oxidized by oxygen, leading to the generation of significant quantities of ROS and, ultimately, cellular damage and cell death. Mitochondria are abundant in cardiomyocytes, making them a prime target for anthracycline accumulation. Compared to other organs, cardiomyocytes do not exhibit higher levels of antioxidant enzymes. These facts combined demonstrate that cardiomyocytes are highly susceptible to ROS damage caused by anthracyclines [50].

### 3.3. Iron Metabolism

Another mechanism by which anthracyclines cause cardiotoxicity is by chelating free iron to form an iron–anthracycline complex. This complex, in turn, reacts with oxygen and promotes ROS production. In addition, anthracyclines interfere with iron metabolism by disrupting iron-binding and iron-transporting proteins. Iron is also sequestered by anthracyclines, interfering with its binding to Iron Regulation Protein-1 (IRP-1). This iron-free portion of IRP-1 promotes transferrin receptor transcription and, as a result, enhances iron uptake by anthracycline-containing cells, particularly the mitochondria-rich cardiomyocytes. In addition to apoptosis and necrosis generated by anthracyclines, the generation of ROS also induces lipid peroxidation and ferroptosis. The latter refers to a recently discovered variant of programmed cell death characterized by the detrimental effects of iron and reactive oxygen species (ROS) on membrane lipids. In support of these findings, recent studies showed that ferroptosis inhibitors could prevent anthracycline-induced cardiotoxicity [50].

### 3.4. Guanylate Cyclase Activity

A wide variety of physiological processes important to the function of endothelial cells and vascular smooth muscle cells as well as cardiomyocytes are regulated by the second messenger cyclic guanosine monophosphate (cGMP). Nitric oxide (NO) and carbon monoxide (CO) both activate soluble guanylate cyclase (sGC), which then produces cGMP. Adverse cardiac remodeling and heart failure have been linked to disturbances in the cGMP signaling system. The NO-sGC-cGMP pathway has emerged as a therapeutic target for cardiopulmonary diseases, including heart failure, myocardial ischemia, pulmonary hypertension, and others. These new therapies, which include cinaciguat, riociguat, and vericiguat, have shown promising results in animal models and are currently being evaluated in a number of clinical trials [51]. Intriguingly, doxorubicin treatment was found to decrease cardiac cGMP levels, and this has emerged as one of the mechanisms of anthracycline-induced cardiotoxicity [52]. Recent studies demonstrated that doxorubicin-induced cardiotoxicity was exacerbated in animal models with reduced cardiac sGC activity [53]. In addition, pharmacological administration of sGC improved left ventricular function in animal models receiving doxorubicin compared to the placebo control, indicating the preventive and therapeutic potential of sGC in anthracycline-induced cardiotoxicity [51]. Therefore, patients with doxorubicin-induced cardiotoxicity would be ideal candidates for these newly discovered NO-sGC-cGMP-targeting medications.

## 4. Factors That May Contribute to Disparities in Anthracycline-Induced Cardiotoxicity

Recently, researchers have paid greater attention to the striking racial disparity in breast-cancer-therapy-induced cardiotoxicity. In the SEER (Surveillance, Epidemiology, and End Results) study, the cumulative incidence of cardiovascular diseases and mortality in breast cancer survivors was significantly greater in African American women (n = 43,562) than in White women (n = 364,025), particularly in those younger than 69 years of age [54,55,56]. Similarly, a large meta-analysis that comprised 18 studies conducted in Europe and North America on anthracycline-treated breast cancer patients revealed that being African American and having cardiovascular risk factors were independent predictors of subclinical and clinical cardiotoxicity [19]. Several other studies highlighted this disparity in anthracycline-induced cardiotoxicity (Table 1). These findings of a notable racial disparity in cardiovascular risk among breast cancer survivors prompted us to investigate possible contributors, including preexisting cardiovascular risk, genetic factors, and social/structural inequalities.

### 4.1. Preexisting Morbidities

The American Society for Preventive Cardiology (ASPC) has recently revised the list of the most significant cardiovascular risk factors. These factors encompass unhealthy dietary patterns; a sedentary way of life; obesity; diabetes; dysregulated lipid metabolism; hypertension; certain demographic characteristics, such as age, gender, and race/ethnicity; smoking; chronic kidney diseases; and genetics/familial hypercholesterolemia. These cardiovascular risk factors will pose a risk for patients receiving cardiotoxic medications, and addressing these risk factors will aid in better management of cancer therapy-related cardiotoxicity [60].

There is a correlation between preexisting cardiovascular risk factors and increased susceptibility to anthracycline-induced cardiotoxicity among breast cancer survivors [10]. According to the ESC guidelines report on cardio-oncology, cancer patients continue to be underdiagnosed and undertreated for a number of cardiovascular risk factors, including obesity, diabetes, dyslipidemia, and hypertension [13]. These comorbidities decrease the overall survival in cancer patients. Therefore, to enhance the long-term outcomes of cancer survivors, it is recommended that these conditions be diagnosed early using standardized risk-based screening and managed according to general ESC guidelines.

African American women diagnosed with breast cancer exhibit a 42% higher mortality rate compared to their White counterparts, primarily attributed to the presence of comorbidities such as cardiovascular and metabolic disorders [61]. Evaluating the modifiable risk variables may help oncologists predict cardiotoxicity, which can help them decide on the type and dosage of cancer treatment, as well as the frequency of post-therapeutic echocardiographic assessment. In a study by Lopez-Neyman et al. [62], the prevalence of cardiovascular disease risk factors, cardiometabolic risk factors, and cardiovascular health metrics across race/ethnicity groups were assessed in 8370 US adults above the age of 20 from the National Health and Nutrition Examination Survey (NHANES) 2011–2018; 55% were African American females. African Americans, regardless of their gender, exhibited a greater incidence of obesity, hypertension, diabetes mellitus, and smoking than other races included in the study. Similarly, nearly half of Mexican Americans had a higher prevalence of obesity and diabetes mellitus when compared to non-Hispanic White people. In this sample of 8370 participants, 55% were African American females, 46.8% were Mexican American, and 49.5% were non-Mexican Hispanic populations.

Individuals diagnosed with metabolic disorders, including diabetes, hypertension, hyperlipidemia, and obesity, have an increased susceptibility to the development of cardiovascular disease, particularly in response to cancer therapy. In support of this statement, previous studies reported a higher development of cardiomyopathy in patients with these risk factors [19,63]. In a study by Qiu et al. [19], the risk of developing cardiotoxicity was measured in a cohort of 7,488 patients who received anthracyclines as monotherapy. They reported a significantly increased risk in patients with diabetes mellitus (OR: 1.74; 95% CI: 1.11–2.74), obesity (OR: 1.72; 95% CI: 1.13–2.61), and hypertension (OR: 1.99; 95% CI: 1.43–2.76).

Cardiovascular risk factors should always be considered before beginning treatment with anthracyclines, especially since cancer patients have an increased risk of developing cardiovascular illness [55,64]. Despite a nationwide decline in cardiovascular mortality, the burden of this disease is heaviest among racial and ethnic minorities [55,65]. Compared to White people, African Americans have an earlier onset and a higher incidence of comorbidities associated with cardiovascular disease (CVD): hypertension, diabetes mellitus, obesity, and hyperlipidemia. In addition, they have a higher incidence of complications, including heart failure, cardiac arrest, cerebrovascular disease, and peripheral vascular disease [55,65]. Inadequate baseline cardiac performance and limited access to primary care are two factors contributing to the higher likelihood of developing cardiac dysfunction in African Americans [64]. Approximately 200,000 preventable fatalities from stroke and heart disease occur annually; African Americans are twice as likely as White persons to die from preventable stroke and heart disease [66].

#### 4.1.1. Obesity

Obesity (body mass index (BMI) > 30 kg/m^2^) is a well-established risk factor for CVD and heart failure. In the 5881 who participated in the Framingham Heart Study, heart failure rates rose by 5% in males and 7% in women for every unit increase in body mass index (BMI) [67]. Body composition and the distribution of fat are also recognized as reliable predictors of cardiovascular risk resulting from cancer therapy [65]. In support of this, animal experiments have shown that a high-fat diet increases the sensitivity to anthracycline-induced cardiotoxicity [68]. In terms of disparity in obesity, African American women were more likely to be obese (58%) than White women (33%) [65,69]. Similarly, the prevalence of obesity in Hispanic individuals (46.2%) was higher than that in non-Hispanic White women (38.7%) per the American Heart Association’s 2021 statistics [70]. It has also been reported that, due to the acculturation process, the prevalence of overweight or obesity among Latina immigrants in the United States was found to be higher among those who migrated at a younger age compared to those who immigrated later in life [71]. Furthermore, the Insulin Resistance Atherosclerosis (IRAS) Family Study revealed that African and Hispanic Americans had larger waist circumferences and visceral adiposity than White Americans [72]. Due to this disparity, Hispanic and African American women are more likely to develop insulin resistance, metabolic syndrome, diabetes, and dyslipidemia, all of which increase their risk of anthracycline-induced cardiotoxicity.

Even when a direct relationship between BMI and overall survival was not significant in some studies, the survival rate was significantly associated with genetic background and socioeconomic factors; these factors were linked to obesity and visceral adiposity in several studies [73,74,75]. Obesity, according to some epidemiological studies, may increase the incidence of hormone-negative breast cancers, reducing patients’ chances of receiving hormonal therapies and leaving them with few options, including anthracyclines, which eventually leads to an increased mortality rate and a poor quality of life [76]. A recent meta-analysis by Guenancia et al. [77] examined the relationship between obesity and overweight and the risk of cardiotoxicity in 8740 breast cancer patients who received anthracyclines. This study demonstrated a robust dose-dependent association between BMI and cardiotoxicity risk; the odds ratio (OR) was 1.15 for overweight patients, 1.47 for obese patients, and 1.38 for both combined. Kabore et al. [78] observed comparable findings in their extensive prospective analysis, whereby they found that obesity exhibited an independent association with an elevated incidence of cardiotoxicity relative to patients with normal weight. At the time of follow-up, patients who were overweight and obese had a 49% and 180% risk of heart failure, respectively.

#### 4.1.2. Type 2 Diabetes

According to the National Health and Nutrition Examination Survey (NHANES), approximately 21.8% of African Americans contributed to the combined prevalence of diagnosed and undiagnosed type 2 diabetes mellitus in the period between 2011 and 2012. Similarly, Hispanic people residing in the United States are 17% more likely to have type 2 diabetes than non-Hispanic White people, according to the Centers for Disease Control and Prevention (CDC). African Americans and Hispanic individuals also experience an increase in the prevalence of prediabetes; this disparity is age-independent and remains high throughout their lifespan [65]. In addition to being several times more likely to develop diabetes mellitus than White women [56,65], minority women have a lower awareness of diabetes, which has contributed to the inability to meet Accountable Care Organization targets (Hba1c < 9%) [65]. It was found that only 54% of African American and Hispanic women accomplish their goals, compared to 61% of White women [65,79]. Therefore, minorities’ susceptibility to cardiac hypertrophy and fibrosis, early-onset diastolic dysfunction, and late-onset systolic dysfunction is increased by prolonged exposure to elevated blood sugar [80].

A meta-analysis by Zhang et al. [81] reported a positive association between type 2 diabetes and the development of cardiotoxicity after the usage of anthracyclines (OR: 1.39, 95% CI: 1.20–1.61, *p* < 0.0001). This study also reported that breast cancer patients with type 2 diabetes were 1.39 times more prone to anthracycline-induced cardiotoxicity than patients without diabetes. Furthermore, findings from the Look AHEAD (Action for Health in Diabetes) study revealed socioeconomically independent racial/ethnic disparities in the initiation of emerging classes of diabetes medications in the United States, such as DPP-4 and SGLT-2 inhibitors and GLP-1 receptor agonists. This disparity was more evident for African Americans, American Indians, and Alaskan Natives [82]. These medications have been shown to improve renal and cardiovascular health; they would have been incredibly beneficial for minorities with a disproportionately high rate of chronic kidney disease and cardiovascular illness [82,83]. In addition, the Look AHEAD study found that annual family income was negatively correlated with the use of newer diabetes medications and that ethnic minorities were more likely to fall into low-income categories with restricted access to more current treatment options [82].

Insulin resistance, a hallmark of obesity and type 2 diabetes, increases the likelihood of anthracycline-induced cardiotoxicity and heart failure [84]. Recent research has revealed that insulin resistance in cardiomyocytes is a predisposing factor to cardiomyocyte apoptosis and cardiac atrophy [85]. A large multicenter study by Gallagher et al. [86] reported a mediation effect between insulin resistance in racial/ethnic minorities and a poor prognosis for breast cancer. In this study, African Americans with invasive breast cancer were found to be more insulin-resistant than White patients (40% vs. 20%); the average homeostatic model assessment of insulin resistance (HOMA-IR) was 1.9 in African Americans versus 1.3 in White patients. Furthermore, a post-surgical Nottingham Prognostic Index (NPI) of more than 4.4 was considered a poor prognostic index and was identified in 28% of African Americans compared to 15% of White patients.

To sum up, much evidence links insulin resistance and diabetes to vascular dysfunction and cardiovascular disease. Therefore, it is likely that the disproportionate prevalence of insulin resistance and diabetes in minority populations contributes to the higher prevalence of anthracycline-induced cardiotoxicity in these communities.

#### 4.1.3. Hypertension

African Americans have a higher prevalence of hypertension, a significant risk factor for cardiovascular diseases. In the United States, 44% of African American women have either diagnosed or undiagnosed hypertension [65]. A comprehensive meta-analysis by Bosco et al. [87] revealed a positive association between hypertension and breast cancer incidence. This study revealed that hypertensive women may have a 15% greater risk of developing breast cancer. This correlation should be considered to guide chemotherapeutic decisions for breast cancer patients. In addition, further research is required to determine the effect of hypertension on anthracycline-induced cardiovascular disease, particularly in the African American population, where hypertension prevalence is known to be higher [61].

Even though hypertension has been well controlled in recent decades, numerous studies have documented racial and ethnic inequalities in hypertension control. A ten-year follow-up of 8796 hypertensive patients by Gu et al. [88] revealed that African American and Hispanic adults have poor hypertension control, as measured by Joint National Committee (JNC) 7 standards: blood pressure of 140/90 mm Hg for patients without chronic kidney disease and diabetes and 130/80 mm Hg for patients with either comorbidity. This difference was more pronounced among African American and Hispanic patients who were younger and uninsured. This study also classified the reasons as patient-related factors, including patient awareness and medication adherence, and healthcare-related factors, including access to a competent healthcare system, patient-centered communications, and physician–patient interaction. Inadequate hypertension management affects both African American and Hispanic patients despite the fact that the former undergo more intense therapy for their condition due to hypertension complications [88].

Minority breast cancer survivors with hypertension and cardiovascular disease have been the subject of scant research to date. Williams et al. [61] investigated the correlation between race/ethnicity and hypertension in breast cancer survivors. This study included 524 participants; 107 were African Americans, and 417 were White. Upon controlling for all confounding factors, it was found that breast cancer survivors of African American descent exhibited a 30% greater prevalence of hypertension (95% CI: 1.11 to 1.52) than their White counterparts. These findings suggest that African Americans have an elevated risk of cardiovascular disease, mainly due to hypertension, despite the effective management of other comorbid conditions, such as diabetes and obesity.

A recent meta-analysis by Zhang et al. [81] listed the risk factors of anthracycline-induced cardiotoxicity from thirteen studies (2544 patients) published between 2008 and 2021. This study found hypertension to be the most significant risk factor for anthracycline-induced cardiotoxicity (OR: 2.95, 95% CI: 1.75–4.10, *p* < 0.0001), followed by concurrent trastuzumab use, coronary artery disease, and the cumulative dose of anthracyclines. Similarly, Kotwinski et al. [89] reported that among 165 women who underwent anthracycline treatment for breast cancer, individuals with slightly elevated baseline blood pressure exhibited an elevated risk of cardiotoxicity. These findings were supported by two investigations in Hungarian and Lithuanian women with breast cancer; these studies demonstrated a significant correlation between preexisting hypertension and the risk of systolic dysfunction and heart failure [90,91]. Together, the findings of prevalent hypertension in minorities and those supporting the association between preexisting hypertension and cardiotoxicity lead us to believe that hypertension may contribute at least in part to the racial and ethnic disparities in anthracycline-induced cardiotoxicity.

#### 4.1.4. Hyperlipidemia

Hyperlipidemia is an additional risk factor contributing to cardiovascular complications in general and is expected to contribute to cardiotoxicity induced by chemotherapy. African American women are more likely than White women to have dyslipidemia (relative risk (RR), 1.17; 95% CI, 1.08–1.28), and this risk increases with age; the RR of dyslipidemia for African American women versus White women is 1.39 (95% CI, 1.00–1.95) in women over 75 years of age [56,65]. African Americans are also at a greater risk of developing atherosclerotic cardiovascular diseases. Agarwala et al. [92] found that the incidence of myocardial infarction among African American women was greater than that of White women and comparable to that of White males over 65. In addition to having a higher risk of dyslipidemia, women from ethnic minorities are less likely to attain optimal lipid targets because they are less frequently prescribed lipid-lowering therapy than White women. It was discovered that medication adherence and drug access varied by geography, healthcare access, socioeconomic status, education level, cultural values, and perceived benefits, all of which are known to vary among people of various racial/ethnic backgrounds [93].

It was discovered that cholesterol levels impart cancer resistance to numerous malignancies, including breast cancer. The increased cholesterol level promotes cell proliferation and survival through expanding lipid rafts. Studies on rodents indicate that hyperlipidemia accelerates tumor development and metastasis. For example, a study by Yun et al. [94] demonstrated how cholesterol regulates cancer sensitivity to doxorubicin treatment. In this study, elevated cholesterol levels were associated with elevated levels of lipid rafts on the cell surface. Cholesterol depletion by statins or gene silencing led to lipid raft internalization, interfering with prooncogenic cell signaling, such as epithelial growth factor receptor (EGFR) and Src kinase, thereby enhancing chemosensitivity and decreasing the dose required to induce cell apoptosis. These data together point to hyperlipidemia as a substantial contributing factor to the racial and ethnic inequality seen in anthracycline-associated cardiovascular complications.

#### 4.1.5. Cardiovascular Diseases

Since 1975, cardiovascular disease has been one of the top two major causes of mortality in the United States, accounting for one in every four fatalities. This condition encompasses cerebrovascular disease, coronary artery disease, aortic atherosclerosis, and peripheral artery disease. In 2015, CVD was responsible for about 423 million illnesses and an estimated 18 million fatalities. The most current national vital data reveal that CVD was the cause of death for 774,165 Americans in 2015, which accounted for more than 29% of all deaths that year. Age-adjusted mortality rates for both stroke and heart disease have exhibited notable declines of 61% and 70%, respectively, since 1975. However, it is important to note that African Americans have not observed similar decreases in mortality rates. In fact, they still face heart disease death rates that are 20% higher and stroke death rates that are 40% higher compared to other racial and ethnic groups within the United States [95].

African Americans and other disadvantaged racial and ethnic minorities have persistent inequities in access to high-quality cardiovascular healthcare, adding to their already-heavy disease burden. Despite the fact that the excess burden of cardiovascular disease in ethnic minorities is largely attributable to adverse clinical, behavioral, and lifestyle-mediated risk factors, it is clear that community and neighborhood factors play a role in this disparity as well [96,97,98]. It has been reported that middle-aged African American women are most affected by this disparity; due to the prevalence of risk factors such as obesity and diabetes, they tend to develop cardiovascular disease much earlier than their White counterparts [99]. In addition, several elements, including genetic, environmental, social, and physical factors, have a crucial influence on the development of cardiovascular diseases.

Epidemiological research suggests that African Americans are at a higher risk of cardiovascular disease due to their higher levels of chronic stress. The latter could be attributed to discrimination at different levels (institutional, structural, interpersonal), poor nutrition, lack of physical activity, and domestic violence. According to a recent study conducted by the Inter-generational Impact of Genetic and Psychological Factors in Blood Pressure (InterGEN), it has been found that parental stress has the potential to alter DNA methylation patterns in African American women. This alteration in DNA methylation may serve as a contributing factor in their susceptibility to hypertension, a significant cardiovascular risk factor. In addition to these factors, risk underestimation and lack of awareness account for the vast majority of disparities [16].

Previous research has indicated that African Americans have a high prevalence of cardiovascular risk factors at the time of cancer diagnosis. For example, a study from the Maryland Cancer Registry found a 33% increase in the risk of cardiovascular mortality among African American women versus White women [100]. Fifty-one percent of the study’s survivors reported hypertension, and although only 7% were obese when they were initially diagnosed with breast cancer, 54% were obese when they were surveyed a few years after their initial diagnosis. Moreover, only 28% of African American breast cancer survivors achieved the level of physical activity suggested by the American Heart Association. Similar to African Americans, Hispanic individuals are disproportionately afflicted by cardiovascular disease, the second leading cause of mortality among Hispanic/Latino people. Hispanic adults bear a disproportionate share of the public health burden of cardiovascular disease, and the American Heart Association has released a scientific statement emphasizing this fact and urging the creation of culturally customized therapies and the inclusion of Latinos in national efforts to promote heart health [101].

While prescribing anthracyclines to patients with preexisting cardiovascular disease is discouraged, cardiovascular risk factors such as obesity, diabetes, smoking, hypertension, and hyperlipidemia have not been taken into account. Likewise, arrhythmias and valvular heart disease have received little attention. With the prevalence of risk factors among minorities, there is a need for dose modifications and an evaluation of risks and benefits to accommodate this population best.

### 4.2. Genetic Factors

**Figure 2:**
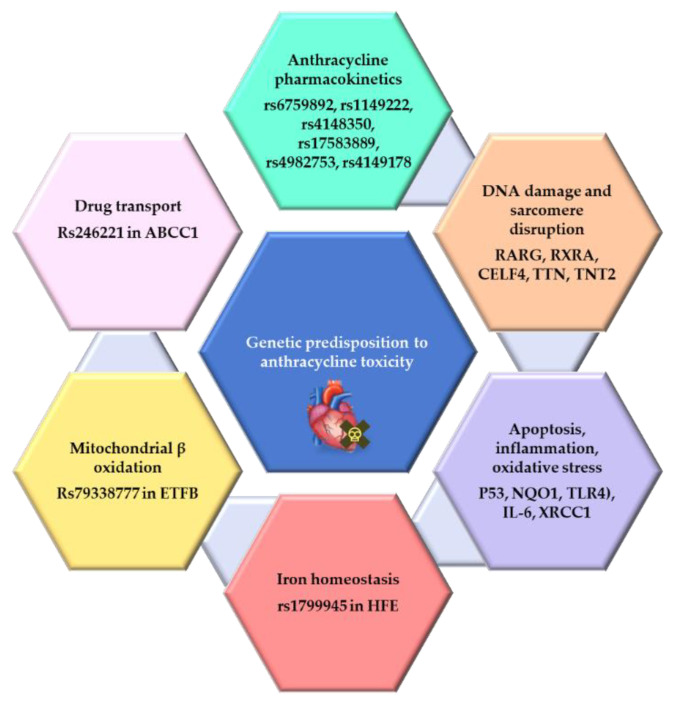
Graphical representation of some of the genetic variants linked to differential toxicity to anthracyclines.

Several clinical risk prediction models have been created, yet their predictive efficacy in identifying breast cancer patients at high risk for anthracycline-induced cardiac dysfunction is lacking [102,103]. Therefore, efforts that integrate clinical risk prediction factors with genetic variants, demographic variables, and polygenic risk factors may be more effective at identifying breast cancer populations at high risk of developing cardiotoxicity in response to chemotherapy. Pharmacogenomics is an emerging discipline investigating individual genetic variations in conjunction with polygenic risk factors that may influence drug efficacy, response, and metabolism [104]. This integration of genetic profiles with drug responses has the potential to yield novel information for risk stratification, treatment customization, and individualization based on a patient’s genetic and clinical characteristics [104]. Some of the genetic variants linked to differential toxicity to anthracyclines are summarized in Figure 2.

As a first step in this direction, Visscher et al. [105] developed a prediction model in pediatric cancer survivors by combining genetic variants of drug metabolism genes with clinical risk factors, classifying patients into three risk groups. Seventy-five percent of patients in the high-risk group developed anthracycline-induced cardiomyopathy, whereas ninety-six percent of patients in the low-risk group did not develop any cardiac dysfunction. Later, the genotyping panel was expanded by adding over 300 genes and over 4500 genetic variants that are relevant to pharmacokinetics and dynamics. This improved model of prediction was able to differentiate between healthy people and patients across several cohorts [106]. However, a similarly effective model for predicting anthracycline-induced cardiotoxicity in breast cancer patients has yet to be developed. Consequently, developing and validating a model that incorporates multiple factors that may contribute to treatment-induced cardiotoxicity is imperative. Once a reliable prediction tool capable of distinguishing high-risk individuals is developed, those with a favorable risk profile may benefit from a higher cumulative dose with a greater anticancer impact. Those with an unfavorable risk profile, on the other hand, would benefit from other therapy choices and more frequent cardiac monitoring, which would allow for the early diagnosis of cardiotoxicity [107].

Breast cancer is classified into three molecular subtypes based on estrogen receptor (ER) and progesterone receptor (PR) status as well as human epidermal growth factor receptor 2 (HER2) expression. These categories are luminal A (ER+/PR+/Her2-), luminal B (ER+/PR+/Her2+), HER2-positive (ER-/PR-/Her2+), and triple-negative (ER-, PR-, HER2-). This categorization is important since each molecular subtype has distinct therapeutic approaches [108]. According to findings from several population-based studies, African American women are more likely to be diagnosed with hormone receptor (HR)-negative or triple-negative molecular subtypes than White women [109,110]. This disparity in molecular subtypes is clinically significant because HR-negative and triple-negative tumors are typically associated with a poor prognosis and do not respond to existing endocrine-based therapies such as tamoxifen and Herceptin, leaving patients with limited treatment options, mainly anthracyclines. In addition, this disparity prompted researchers to postulate genetic risk factors that place African American women at a greater risk of developing HR-negative breast cancer [111]. This hypothesis is supported by the fact that most women with HR-negative breast malignancies carry BRCA1 mutations [112]. It was shown that Hispanic women, like African American women, had a greater chance of developing estrogen-receptor-negative cancers than non-Hispanic White women. Hispanic women had an unusually high rate of HER2-positive tumors and a lower likelihood of the more favorable luminal A subtype, but no differences in the triple-negative subtypes were detected [113].

There is emerging evidence that genetics plays an essential role in how different populations respond to treatments, including chemotherapy. One of the most prevalent genetic reasons for variable responses to treatment among individuals from diverse racial/ethnic origins is single-nucleotide polymorphisms (SNPs). SNPs are variations in the DNA sequence present in at least 1% of the population and caused by a nucleotide’s deletion, insertion, substitution, or duplication [114]. Several studies have shown that SNPs and other genetic factors may influence the differential susceptibility to cardiotoxicity among diverse individuals. These changes may be attributable to SNP-mediated modifications in proteins implicated in the drug’s absorption, elimination, intracellular transport, metabolism, and cytotoxic/apoptotic effects on non-tumorigenic cells [114,115]. Consequently, identifying specific genetic variants and their molecular markers, which make breast cancer patients or survivors susceptible to cardiac dysfunction, is essential for cardiotoxicity risk stratification [116].

Several gene variants, including rs6759892, rs1149222, rs4148350, rs17583889, rs4982753, and rs4149178, were found to be associated with anthracycline absorption, metabolism, and elimination, and they demonstrated direct association with anthracycline-associated cardiovascular dysfunction [117]. In a study of 877 breast cancer patients, heterozygous carriers of the rs246221 T-allele in the ABCC1 gene, which is involved in the energy-dependent transport of cytotoxic agents out of the cell, had a greater risk of decreased left ventricular ejection fraction (LEVF) following chemotherapy treatment than homozygous carriers of the T allele [118]. In a breast cancer survivor cohort from Spain, a missense variant (rs79338777; Pro52Leu) in the electron transfer flavoprotein (ETFB) gene, which is involved in mitochondrial β-oxidation and ATP production, was associated with an increased risk of chronic anthracycline-induced cardiac dysfunction [119]. Also, a cohort of Lithuanian breast cancer patients revealed that the rs1799945 H63D variant of the homeostatic iron regulator gene, HFE, results in the substitution of aspartate for histidine at position 63 and is associated with anthracycline-induced cardiotoxicity [120]. Similarly, a polymorphism in the carbonyl reductase (CBR) gene (CBR3 V244 variant), which catalyzes the reduction of anthracyclines to cardiotoxic alcohol derivatives, was associated with a decline in LVEF in breast cancer patients following anthracycline treatment [121]. Genome-wide association and pathway analysis in a cohort of 84% White breast cancer patients who received anthracyclines revealed that the presence of SNP rs10443221 on chromosome 1 (1p32.1) resulted in a 4.11-point increase in LVEF for each alternate allele, thereby conferring protection against anthracycline-induced cardiotoxicity [122].

Previous studies investigated how SNPs in genes involved in apoptosis, inflammation, oxidative stress, DNA damage, and mitochondrial dysfunction modify the response to anthracyclines [123,124,125,126,127,128,129]. Some examples of these genes included the tumor suppressor gene, P53, NADPH quinine oxidoreductase 1 (NQO1), toll-like receptor 4 (TLR4), interleukin6 (IL6), and X-ray repair cross-complementing group 1 (XRCC1). Breast cancer patients with the wild-type form of P53 Arg72Pro, which promotes apoptosis more efficiently, were more sensitive to anthracyclines than those homozygous for proline [128,130]. Among African Americans, the prevalence of the homozygous Pro72 allele was 17%, whereas it was only 7% among White individuals, partially explaining the resistance observed in the former population [131]. Another example is the Pro187Ser polymorphism in NQO1, which causes the significant inhibition of enzyme activity. Breast cancer patients with a homozygous mutation in NQO1 displayed diminished sensitivity to epirubicin and poorer survival rates [129,132]. Previous studies have shown that women of African American descent are more likely to have two TLR4 loss-of-function polymorphisms, Asp299Gly and Thr399Ile, which have been linked to immune evasion and early recurrence in breast cancer patients treated with anthracycline-based chemotherapy [126,133]. Similarly, genetic variations (174 G/C) in the proinflammatory gene IL6 were more prevalent in African Americans than in White individuals. This polymorphism is associated with higher circulating levels of IL6, chemotherapeutic resistance, and lower disease-free survival in breast cancer patients receiving anthracyclines [127,134,135]. Finally, the XRCC1 Arg399Gln polymorphism (rs25487), which affects its DNA repair capacity, was found to be prevalent among patients of African ancestry and was associated with decreased survival in these patients following anthracycline administration [125,136].

In addition to genetic variants that influence pharmacokinetics or pharmacodynamics, disparities in the tumor microenvironment, the local compartment where cancer cells reside, play a significant role in the racial disparity in breast cancer outcomes between White and African American patients [137]. The tumor microenvironment comprises numerous cell types, including immune cells, endothelial cells, adipocytes, fibroblasts, and extracellular matrix components. This local niche influences the behavior of cancer cells by secreting chemokines, cytokines, and growth factors that interact bidirectionally with tumor cells [137]. There is evidence that the microenvironment of a tumor may be linked to racial disparities. African Americans have more pro-tumorigenic immune cells, such as tumor-associated macrophages and lymphocytes, than their White counterparts. They also have higher levels of inflammatory cytokines, which help create a pro-metastatic tumor microenvironment. Also, tumor beds in breast cancer patients of African descent showed more microvasculature and distorted vessels. These immune cell density and angiogenesis increases could be attributed to evolutionary selection for a more robust immune response in African ancestry [138]. Collectively, these changes result in more aggressive, treatment-resistant phenotypes in African American patients, which may necessitate higher dosages and ultimately increase cardiotoxicity.

### 4.3. Socioeconomic Determinants

Although strides have been made in breast cancer treatment and cardio-oncology, African American and Hispanic women are disproportionately affected by cardiotoxicity and cardiovascular-related comorbidities compared to their White counterparts [10]. This inequality may be attributed, at least in part, to disparities in the socioeconomic determinants of health experienced by each group. According to the World Health Organization, social determinants of health include a wider range of factors and systems that influence the necessities of daily living in addition to the circumstances under which people are born, grow, work, live, and age [18]. There is a disproportionate load of illness among African Americans, low-income communities, and other minority groups [11]. The observed health disparities involve a complex interaction of the following domains: biology, behavior, physical and socio-cultural environments, and the healthcare system; these disparities are influenced at individual, interpersonal, community, and societal levels [11,17]. While these disparities have been continually affecting racial and ethnic minority groups, they have been amplified by the recent COVID-19 pandemic [11,139,140]. Even though they can be prevented or easily managed by medications, the comorbidities are less likely to be controlled in racial/ethnic minority groups, particularly in younger demographics [140].

#### 4.3.1. Education and Socioeconomic Status

Socioeconomic status (SES) is determined by variables such as education, occupation, income, and residential neighborhood [55]. There are disparities in cancer incidence, mortality, and survival between patients with a lower SES and those with a higher SES. Low-SES individuals in underserved areas typically have higher rates of late-stage diagnosis than individuals from affluent or high-SES areas [141]. Social class, race, and ethnicity all have entangled relationships with one another. The socioeconomic level of a person is typically determined by their race and ethnicity. Additionally, neighborhoods are often divided along racial, cultural, and socioeconomic lines. Low socioeconomic status (SES) has been continuously linked to a plethora of social issues, including underdevelopment in economic opportunity, poor health, and inadequate educational achievement.

Compared to the 14% poverty rate among non-Latino White and Asians, the rates of poverty among African Americans and Latinos in the United States are much higher: 39% and 33%, respectively. Even after controlling for molecular subtypes and cancer stage at diagnosis, women from minority racial/ethnic backgrounds have lower breast cancer survival rates than White adults [141,142]. Lack of health insurance and restricted access to medical treatment and mammography stand out as contributing factors to the lower survival rates in the former group [141]. These aspects depend on the SES, which is determined by a person’s education level, occupation, and income, all of which are somewhat interdependent [55]. The findings of a retrospective study including a sample size of 79,167 individuals who had previously been diagnosed with breast cancer revealed that African Americans had a higher susceptibility to cardiovascular disease in comparison to individuals of White ethnicity [143]. In comparison to White patients, members of racial minorities are more likely to live in multidimensional poverty, and they also have 50% higher mortality from breast cancer [141,144].

#### 4.3.2. Unequal Healthcare Access

Low-quality treatment, insufficient access, difficulty navigating the system, and provider ignorance or prejudice are all possible causes of racial and ethnic disparities in general health. Avoidable surgeries, unnecessary hospital stays, and untreated diseases have all been linked to socioeconomic level and race/ethnicity. There are major discrepancies in insurance protection across different races. Health insurance coverage is much lower among Hispanic people and African Americans in preretirement compared to White and Asian individuals. Accessing healthcare requires having health insurance, access to transportation, and proximity to an appropriate healthcare facility [55]. Patients, particularly those of lower SES or with preexisting chronic conditions, may be unable to find reliable transportation to places with specialists to adequately address cardiotoxicity [55,145]. Furthermore, many cancer patients do not have health insurance, and even when they do, access to cardio-oncologists is not guaranteed, as not every hospital in the United States employs cardio-oncologists. Therefore, inadequate insurance coverage, lack of access to transportation, and difficulty accessing cardio-oncologists put significant pressure on patients [55]. Another point to consider is the higher cost of less cardiotoxic treatment therapies [55]. There are decreased rates of clinical and subclinical cardiotoxicity and heart failure with novel approaches such as liposomal doxorubicin and continuous infusions of anthracyclines. However, these relatively novel interventions are more costly and largely unavailable to populations with lower incomes [55,146,147].

The unequal healthcare access problem is also caused by ineffective institutional policies. Systemic prejudice is not anomalous conduct exhibited by a limited number of individuals but rather frequently reinforced by institutional rules and unconscious biases rooted in negative stereotypes. To mitigate the rising expenses and enhance healthcare accessibility, State health agencies implemented managed care in their Medicaid program. In states where Medicaid was expanded under the ACA (American Care Act), uninsurance rates have decreased dramatically among community-healthcare-enrolled patients. The rate of uninsured visitors to CHC in Medicaid-expanded states has dropped by 57% after the ACA introduction compared to only 20% in non-expansion states [145]. From these data, one can conclude that states with expanded Medicaid can experience larger reductions in healthcare disparities in cancer patients and survivors. This will ensure timely healthcare access for these populations and prompt a reduction in complications such as cardiotoxicity due to more aggressive treatment. Perhaps the greatest benefit of Medicaid expansion could come from access to preventative care. More investigations are needed to shed light on the degree of change in healthcare disparity following the ACA introduction.

Nevertheless, these policies are not without limitations. For example, the lower payment rates offered by Medicaid in comparison to commercial insurance can act as a deterrent for certain healthcare providers, leading them to be less inclined to accept Medicaid patients. Consequently, this exacerbates the scarcity of available healthcare providers for geographically isolated individuals who rely on Medicaid coverage [148]. Furthermore, managed care organizations seek physicians that demonstrate cost-effective practices by minimizing the number of operations ordered, prescribing medications judiciously, and limiting referrals [149]. On the other hand, minority and low-income populations often have more severe disease presentations upon diagnosis. Consequently, these individuals necessitate comprehensive management, which may entail increased utilization of medical services that are not guaranteed in this system. Therefore, considering that managed medical care became our nation’s safety net that is designed to provide care to patients of low income, it is imperative to comprehensively explore the efficacy of managed care policies in mitigating racial disparities in healthcare access and utilization.

#### 4.3.3. Underrepresentation in Clinical Trials

Despite initiatives taken by federal agencies, the recruitment of minority groups to clinical trials is poor [150]. The analysis of 230 studies conducted for the purpose of obtaining US Food and Drug Administration (FDA) approval for oncologic treatments revealed that African American individuals accounted for a mere 3% of the total patient population of 100,000. However, it is worth noting that this figure indicates a positive shift in this pattern compared to the data recorded in 1985 [150,151,152,153]. Despite making up only 60.7% of the US population, according to the 2018 US Census Bureau estimates, non-Hispanic White people of European descent comprise more than 90% of those participating in clinical trials [154]. Since there are race- and ethnicity-related disparities in the biology of tumors, it is imperative that minority populations be represented in clinical trials [150]. Merely 21% of the cohort studies and clinical trials focused on cardioprotective medicines included demographic information pertaining to the race, ethnicity, socioeconomic status, or other social determinants of health of the participants. Because of this imbalance in clinical research inclusion, the application of clinical data, therapeutic indices, and the safety and toxicity of drugs have been limited. Other repercussions include ineffective screening and treatment of cancer due to genetic and other biological variations. Consequently, it is crucial to address this issue, as underrepresentation obscures the health problems encountered by racial and ethnic minority groups [150].

#### 4.3.4. Housing and Food Insecurities

Housing insecurity is a major social determinant affecting the treatment and survival of a disease. According to the biobehavioral theory of health, home instability is a factor that contributes to food insecurity at both environmental and emotional levels. Inadequate housing is a barrier to healthcare that causes patients to delay, miss, or cancel their doctor appointments. This, in turn, increases hospitalization rates among low-income groups [155]. A qualitative study conducted among patients with cancer and cancer survivors in New York identified twenty-nine distinct housing-related issues, including housing affordability, homelessness, living conditions, housing quality, accessibility, and security [156]. Cancer patients’ susceptibility to housing insecurity in low-income communities was associated with reduced cancer survival rates. In addition, these insecurities were linked to a higher prevalence of coexisting medical diseases, including diabetes, hypertension, hyperlipidemia, cardiac conditions, and medication non-adherence [156]. On the other hand, a population-based study by Gupta et al. [156] found that patients with positive home equity following a cancer diagnosis were more likely to get treatment, increasing their likelihood of survival.

Food insecurity is another disparity that stands as a barrier to healthcare access and health. Nearly 35 million Americans experience food insecurity—a lack of access to adequate or quality food for an active, healthy lifestyle [157]. The prevalence of this phenomenon exhibits a greater proportion within racial and ethnic minority groups, single-parent and low-income households, and households characterized by lower educational attainment [158]. Research has shown the connection between food insecurity and chronic medical conditions [158,159,160]. Additionally, several population-based studies have suggested that those residing in homes experiencing food insecurity may be more likely to develop cancer. The United States Department of Agriculture (USDA) has also discovered that the level of food insecurity correlates with an increase in cancer prevalence, with 3.9% of low-income families reporting cancer compared to 5.8% of households with very low food security. The correlation between the diagnosis of cancer and food insecurity remains significant even after controlling for several socioeconomic variables. A research study by Gany et al. [155], which reported a 55% prevalence of food insecurity in underserved, low-income, multiethnic minority groups receiving cancer treatment from New York City clinics, also supports this claim.

#### 4.3.5. Environmental Safety

The environment may play a significant role in exacerbating the side effects of chemotherapeutic agents in patients who reside in areas with high levels of air pollution. The enhanced generation of reactive oxygen species (ROS) and the induction of prothrombotic and proinflammatory signaling are some of the mechanisms by which ambient air pollution can exacerbate cardiotoxicity in already-compromised patients [161]. Some ultrafine particles can infiltrate the bloodstream upon inhalation and disrupt endothelial function by triggering an inflammatory response. The induction of ROS-related effects presents another potential route for causing direct damage to the heart muscle. It has been discovered that contaminants linked to urban traffic, including organic and elemental carbon, carbon monoxide (CO), lead (Pb), iron (Fe), nitrogen dioxide (NO2), and others, considerably enhance the risk of cardiovascular death [161].

Environmental contaminants such as cadmium and lead have been recognized as significant coronary risk factors. Lead-induced oxidative stress is caused by the production of hydroxyl radicals. Moreover, by mimicking calcium, lead can be transported in endothelial cells and assume some of the calcium’s normal functions, resulting in decreased nitric oxide (NO) bioavailability and the development of hypertension [162]. Similar to lead, cadmium may restrict NO production by inhibiting endothelial NO synthase activity. Cadmium can also replace zinc, interfering with calcium signaling and zinc-dependent antioxidant enzymes, leading to a decrease in NO production and free radical scavenging [162].

Lack of access to clean drinking water is another factor that can negatively impact low-SES communities. Chronic exposure to arsenic in potable water is a significant factor in cardiotoxicity. Environmental sources like contaminated well water, occupational sources like pesticides, and medicinal sources like arsenic trioxide used to treat acute promyelocytic leukemia are significant sources of arsenic exposure [163]. Several mechanisms have been identified for arsenic-induced cardiotoxicity, including oxidative stress, interference with cardiac ion channels, DNA fragmentation, and apoptosis. Arsenic-induced cardiotoxicity can also occur via ROS production and mitochondrial disruption, activating the intrinsic apoptotic pathway via the release of cytochrome C and activating the effector caspases, particularly caspase 3 [163]. Additional pollutants and volatile organic compounds (VOCs) can increase the risk posed by air pollution. However, there is a lack of valid testing for some of these pollutants, which poses an additional health risk and necessitates progress in evaluating the entire continuum of toxic substance exposure, particularly in patients undergoing systemic chemotherapies.

#### 4.3.6. Structural Racism and Violence

Systemic racism can have devastating effects on the early detection and diagnosis of several cancers, including breast and lung cancers. Inequality in treating early-stage breast cancers has been documented in African American patients compared to their White counterparts. Poor communication, mistrust, and implicit bias are factors associated with inconsistencies in treatment [164]. Accountability for Cancer Care through Undoing Racism and Equity (ACCURE) is a 5-year study exploring the interventions on racial disparity in early-stage lung and breast cancer treatment between African American and White patients. The primary outcome, as it pertains to the breast cancer arm of the study, was surgery (mastectomy), radiation, or four cycles of chemotherapy to be completed. In the overall model, it was shown that patients lacking private insurance had comparatively lower rates of treatment completion. The results of the multivariable analysis indicated a lower rate of treatment completion among African American patients in comparison to White patients in both the retrospective and concurrent control groups [164]. A system-based, multifaceted, practical approach to intervention can improve treatment completion for African American patients in the early stages of lung or breast cancer and reduce racial disparity in concurrent and historical controls.

One of the greatest obstacles in structural-racism-related research is determining when race plays a role in early treatment bias. A systematic review of 52 relevant breast cancer studies revealed that formal definitions of racial and ethnic variables are either lacking or ambiguous. In breast cancer disparity research, the lack of a precise conceptualization of race and failure to recognize various forms of racism remain obstacles [165]. In order to reduce racial health disparities, it is necessary to strengthen methods for reporting systemic racism. The combined emotional and psychological stress of multiple forms of violence can have devastating effects on the treatment and outcome of affected breast cancer patients. A common form of violence against women is domestic violence. In a literature review, ten articles published between 2005 and 2014 reported abusive behavior toward women dealing with some forms of cancer. Factors that place one at a higher risk of experiencing this form of violence include a history of physical or psychological abuse, substance abuse, young age, instability and economic stress, marital conflict, and poverty [166]. The effects of this abuse are widely spread, and symptoms can be challenging to determine if the women are undergoing cancer treatment. For example, domestic violence results can include anxiety, post-traumatic stress disorders, chronic pain, gastrointestinal issues, unintended pregnancies, etc. An estimate shows that 1 in 8 women will be diagnosed with breast cancer, and 1 in 4 will have experienced domestic violence [166].

Living in neighborhoods characterized by high crime levels, gun violence, and other aspects of violence causes significant psychological stress; this stress has been associated with poor therapeutic responses in cancer patients [167]. The oscillations in stress hormones, such as glucocorticoids and catecholamines, which are regulated by the sympathetic nervous system and the hypothalamic–pituitary–adrenal axis, influence an individual’s psychological stress reactions. The activation of these systems sends signals to a range of processes involved in cell growth, differentiation, and immune responses. In addition, psychological stress induces a multitude of cardiometabolic reactions, such as vasoconstriction, tachycardia, and catabolic signaling pathways, all of which are anticipated to increase the risk of cardiotoxicity in cancer patients [168]. Furthermore, in chronic stress, immune signaling is altered toward a proinflammatory state due to aberrant crosstalk between the neuroendocrine and immune systems. In multiple types of cancer, including breast cancer, resistance to therapy has been linked to stress-induced chronic low-grade inflammation and immune evasion by cancer cells [169].

A study by Eldridge and Berrigan [170] demonstrated that women residing in neighborhoods characterized by elevated levels of structural racism in domains such as educational achievement, judicial integrity, and political engagement face an increased susceptibility to developing breast cancers that exhibit greater aggressiveness and resistance to treatment, specifically triple-negative breast cancer (TNBC). Compared to White women who lived in the same neighborhoods, the likelihood of breast cancer was even more remarkable in African American women. The odds ratio for the diagnosis of TNBC in women living in regions with high structural racism compared with those in areas with lesser structural racism was 1.50 for African American women and 1.17 for White women. These results provide credence to the idea that placing racial health inequalities within their appropriate context is necessary. Additional investigation is warranted to examine the underlying mechanisms through which institutional racism exerts its influence on disparities in health outcomes. We risk worsening cancer disparities if we do not recognize the broader determinants of health and systemic disadvantage of marginalized groups and respond to them structurally rather than individually. Once structural violence and racism have been properly identified in the oncology clinic setting, women could be given opportunities to voice their experiences and help clinicians better understand the complexity of racial disparity in treatment response.

#### 4.3.7. Patient Advocacy and Community Interventions

Patient advocacy is primarily portrayed by individuals who belong to the upper-middle class and/or are of White ethnicity. These individuals are extensively showcased in numerous media outlets, including television interviews. Patients who advocate for the accessibility of health data tend to possess higher socioeconomic advantages in comparison to persons who live in areas impacted by digital redlining or those who lack the financial resources to afford mobile devices or smartphones for accessing essential health information [171]. Despite being in a demographically highly diverse nation, our cultural framework has predominantly revolved around norms and conventions that cater to the majority population. This phenomenon is also evident within the healthcare system. Medical schools now lack the curriculum and emphasis on training healthcare personnel to possess the essential cultural competencies and preparedness required to effectively evaluate the culture-specific values of their minority patients during interactions. The existence of such barriers hinders the comprehension and rapport between patients and healthcare providers. Addressing healthcare disparity is a complex issue that lacks a straightforward solution. However, the inclusion of patient advocates who possess the necessary educational background and cultural diversity has the potential to mitigate the gaps in healthcare inequality. In addition, community-based interventions that focus on population-level prevention have the potential to effectively reduce the prevalence of health risk factors among communities of color, hence addressing the existing healthcare disparities gap [172].

Collectively, despite the fact that the relationship between poor therapeutic outcome and race/ethnicity may not be understood by conventional means, it appears that multiple forms of inequity, both biological and non-biological, contribute to the disparate response to anthracycline treatment among patients from minority backgrounds. This concept is known as “*intersectionality*” and describes how personal characteristics intersect with systems and structures to influence an individual’s experience [173]. The investigation of health disparities has traditionally focused on a single dimension, such as race, gender, or socioeconomic position. However, there has been a growing recognition of the need to use intersectional approaches in order to comprehensively analyze the cumulative health impacts of several social inequalities. For example, and in accordance with the intersectionality perspective, empirical research has shown that the health disparities experienced by African American women exceed the cumulative effects of both racial and gender disadvantages [174]. Another research study has provided evidence indicating that socioeconomic resources, such as education, income, and wealth, offer relatively less protection for the health of African American individuals in comparison to their White counterparts [175]. Hence, it is imperative to adopt future multidimensional methodologies that encompass variables such as gender, social class, and economic status in order to comprehensively examine racial and ethnic disparities.

## 5. Lifestyle Factors

Healthy behavior and lifestyle enhance the general health status of the population and aid in preventing chronic diseases. The regular consumption of fruits and vegetables and physical activity are inversely correlated with cardiovascular disease, diabetes, numerous types of cancer, and overall mortality. Indeed, excessive caloric intake relative to a person’s daily requirements could result in obesity and all of its consequences, a prevalent problem in urban societies. The consumption of fast foods with low nutritional values is becoming progressively more common [176]. In addition to poor dietary practices, inactivity is the fourth leading cause of mortality around the globe. As we discussed above, lifestyle-related morbidities are known to predispose patients to the adverse effects of chemotherapy. It is essential to identify ethnic differences in adopting unhealthy lifestyles to comprehend racial/ethnic disparities in chemotherapy-induced cardiotoxicity [177].

The BMI levels of adolescents and adults serve as an early indicator of this racial/ethnic lifestyle disparity. A study of 7956 children aged 3 to 11 revealed that African American and Hispanic youth were more likely to develop obesity than White children. Time spent in front of video games, improper dietary habits, a lack of sleep, and insufficient physical activity are the most prevalent behaviors among African Americans and Hispanic people compared to their White counterparts, which explains the BMI gap. In addition, African American and Hispanic individuals of all ages were more likely than White individuals to consume more than two sugar-sweetened beverages (SSBs) per day, to consume high-calorie fast food, to skip breakfast more than once a week, and to eat family meals fewer than five times per week [178].

Discrepancies in the types of nutrients ingested were observed among different racial/ethnic groups. An initiative funded by the National Institutes of Health to screen 9803 individuals of African American (n = 561), White (n = 7817), and Hispanic (n = 1425) heritage for dietary habits and fat intake revealed that White individuals consumed the least fat among the three groups, after adjusting for several variables, such as age, gender, education level, and employment status. In addition, White participants were more likely to report using low-fat alternatives, avoiding fried and processed foods, and substituting meat proteins. Hispanic participants were more likely to consume vegetables and fruits and avoid cholesterol. African American participants had the lowest likelihood of substituting meat or avoiding high-fat diets. Lastly, African American and Hispanic participants were less likely to peruse food labels and make food selections based on nutrition information on these labels.

Whole grains, essential for general health maintenance, were also consumed differently among different racial/ethnic groups. Their unique bioactive components (protein, dietary fiber, polyphenols, and alkaloids) have antioxidant effects by removing free radicals, modulating blood sugar and lipids, and restoring the diversity of gut flora, among other functions. Carolyn Gugger et al. [179] discovered that the average consumption of whole grains was greatest among non-Hispanic White participants (1.0 oz eq/d), followed by Hispanic individuals (0.7 oz eq/d) and African Americans (0.8 oz eq/d). Similarly, various racial/ethnic groups consumed varying amounts of fiber, which is recommended for maintaining healthy body weight and gastrointestinal function, as well as preventing type 2 diabetes and cardiovascular disease. According to a 2014 survey conducted by the United States Department of Agriculture, African American people consume the least amount of fiber (13 g/day), followed by non-Hispanic White (16 g/day) and Hispanic (17 g/day) [180] individuals. This trend was also confirmed by the most recent available data on dietary fiber consumption provided by the USDA [181], reporting the highest consumption of dietary fiber in Hispanic participants (9.2 g/1000 kcal), followed by White participants (7.7 g/1000 kcal) and African Americans (7 g/1000 kcal).

A 2017–2018 survey on meals eaten outside the home revealed that non-Hispanic African American adults ingested the most calories from fast food (17.4% of daily calories; 281 kcal/day). The same was valid for African American youth, who consumed the highest fast-food-based calories per day (526 kcal/day) compared to youth of other ethnicities [182]. Regarding proteins, 69 percent of the protein consumed by Americans, in general, is derived from animal sources. According to the Third National Health and Nutrition Examination, individuals of different races and ethnicities consume proteins from diverse sources. Hispanic people consumed more protein from eggs and plants, while African Americans consumed more protein from poultry and pork. Conversely, White people ingested more cereal proteins than other racial/ethnic groups [183]. Fruit and vegetable consumption appears to be associated with neighborhood socioeconomic status (NSES), including the quality and quantity of grocery shops that determine the availability of fresh produce and healthy food. Escarce et al. [184] compared the number of daily fruit and vegetable servings consumed by residents of distinct NSES neighborhoods. Neighborhoods with lower NSES were predominantly populated by African Americans with lower incomes, education levels, and fruit and vegetable consumption than White individuals.

A healthy diet and nutritional habits may offer some protection for the cardiovascular system against the harmful effects of chemotherapy drugs. Zhang et al. [185] conducted a comprehensive systematic analysis encompassing 57 research involving both animal and human subjects. The primary objective of these studies was to investigate the potential preventive effects of the diet in mitigating chemotherapy-induced cardiotoxicity. Seven dietary nutrients were found to alleviate chemotherapy-induced myocardial oxidative stress and left ventricular dysfunction. Allicin (found in garlic), lycopene (found in tomatoes), polyphenols (found in green tea, grapes, and other fruits), amino acids (primarily glutamine), coenzyme Q10, polyunsaturated fatty acids, and trace elements (especially zinc and selenium) were among these nutrients. Several additional studies confirmed the significance of healthy nutrition in reducing the risk of long-term cardiovascular complications in chemotherapy-treated cancer patients [106,186]. A study by Byrd et al. [187] reported the adherence to US dietary guidelines of female cancer survivors of different ethnicities. Hispanic women, compared to their White counterparts, were found to be more compliant with fiber consumption guidelines. In addition, Hispanic women had the highest average intake of vegetables, fiber, and calcium. African American women were the least compliant with vegetable guidelines and were more likely to be overweight or obese.

In addition to healthy dietary habits, physical activity has also been shown to have positive effects on women who have survived breast cancer. A study of women’s quality of life (QOL) two years following breast cancer diagnosis found that following the public health guidelines for physical exercise increased vitality and social functioning and improved their overall psychosocial status [188]. Reportedly, ethnic minorities have usually adopted less healthy exercise behaviors than White persons, especially among adults. A study by Patel et al. [177] analyzed physical activity levels among different racial/ethnic groups in the US population. White individuals were considerably more likely to engage in physical activity than Hispanic (40%) and African American people (38%) among 296,802 participants. Stratifying for age, African Americans aged 45 to 64 were significantly less likely to exercise than other racial/ethnic groups [177,189]. Similarly, August and Sorkin’s study of 33,189 Californian adults aged 45 and older found that individuals belonging to racial/ethnic minority groups had lower levels of engagement in moderate or vigorous exercise than White subjects; these disparities were observed to be more prominent toward middle adulthood [189].

According to findings from the National Physical Activity and Weight Loss Survey, there was a notable discrepancy in the age-adjusted frequency of leisure-time inactivity between different racial and ethnic groups. Specifically, the survey revealed that the percentage of White men and women who reported being inactive was lower (9.9% and 12.0%) compared to African American men and women (19.0% and 25.2%) and Hispanic men and women (20.9% and 27.3%). Leisure-time inactivity was greater among lower social class participants in each racial/ethnic group and adjusting for social class eliminated this disparity. Powell et al. [190] found that communities with a higher proportion of African American individuals were significantly less likely to have parks, swimming pools, green spaces, or public beaches than Hispanic and White communities, providing additional evidence for the relationship between physical activity and socioeconomic status (SES).

Tobacco use is a significant risk factor for cardiovascular disease and, by extension, anthracycline-induced cardiotoxicity. Smoking is expected to exacerbate the adverse effects of anthracyclines since it causes oxidative stress and inflammation. Extensive smoking history is a well-recognized risk factor for heart failure, peripheral arterial disease, venous thromboembolism, atrial fibrillation, and sudden cardiac arrest. Smoking cessation has been found to minimize the cardiovascular risk, but it takes more than 15 years of smoking cessation for the cardiovascular risk to equal that of nonsmokers. Notably, this reduction in cardiovascular risk does not apply to those who have been heavy smokers (32 pack-years) for several years [191]. Therefore, previous or current smoking status is an essential factor affecting cancer patients’ susceptibility to cardiotoxicity. In the United States, smoking causes nearly 480,000 deaths per year. A study by Nguyen-Grozavu et al. [192] found a correlation between smoking behavior and race/ethnicity, with African Americans having the highest prevalence. This group begins smoking at a younger age and typically smokes for extended periods before quitting. The findings of a population-based cohort study conducted on African American breast cancer survivors revealed a significant association between smoking at the time of cancer diagnosis and an elevated risk of all-cause mortality [193]. This risk is much higher in women with increased pack-years of smoking. Despite the lack of research that has explicitly investigated the impact of smoking on anthracycline-induced cardiotoxicity, several studies, including The Carolina Breast Cancer Study and a pooled study of 10,000 breast cancer survivors, have indicated worse survival for current and past smokers, particularly among African American breast cancer survivors [193,194,195,196].

In summary, several studies have shown that minority patients had a greater mortality rate from breast cancer when compared to White people. According to these studies, the mortality rate was made worse by several factors, including poor lifestyles, lack of access to appropriate healthcare, lower educational and neighborhood socioeconomic levels, and a history of smoking, all of which are interrelated factors and were found to be associated with racial minority status [194]. It is worth mentioning that epidemiological research and clinical trials investigating the impact of adopting a healthier lifestyle on improving survival or quality of life (QoL) in breast cancer survivors from underrepresented and minority groups are lacking.

## 6. Conclusions and Recommendations

**Figure 3:**
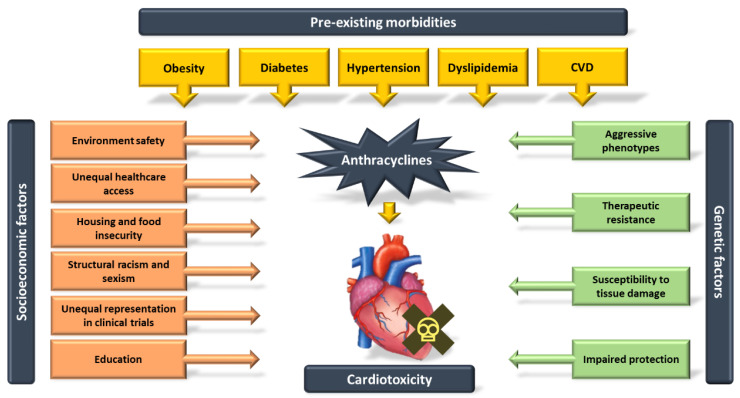
A visual representation of some of the major factors contributing to racial/ethnic disparities in anthracycline-induced cardiotoxicity.

Despite advancements in early detection and effective breast cancer treatment, an increasing number of breast cancer survivors are at risk of developing cardiovascular disease or dying due to treatment-related cardiotoxicity. It is important to note that not all women are exposed to the same levels of cardiotoxicity from breast cancer treatments and that some women from marginalized populations have a higher risk of developing life-threatening cardiac dysfunction than others. As a result, developing risk prediction models to evaluate the likelihood of unfavorable cardiac outcomes among breast cancer patients is an immediate need. To this day, neither the design nor the performance nor the methodological rigor of the risk prediction models that have been constructed has been evaluated in a systematic manner. Furthermore, it is imperative for these models to consider racial and ethnic differences, along with the causes that might contribute to the emergence of such inequities (Figure 3).

While there is no simple solution to the racial disparities in anthracycline-induced cardiotoxicity, adopting a multidimensional approach that takes into account several aspects, such as patient circumstances, as well as aspects related to healthcare providers and the healthcare system, has the potential to mitigate this gap. This approach should incorporate risk assessment as a means to avoid the premature discontinuation of treatment for patients who may derive prolonged therapeutic benefits and mitigate the occurrence of severe consequences in patients who are at a greater risk of developing cardiac toxicity. The findings of these assessments pertaining to cardiovascular toxicity should be effectively conveyed to the patient as well as other relevant healthcare practitioners. In addition, it is imperative to promptly refer patients at high risk to cardiology in order to engage in a comprehensive evaluation of the risk-to-benefit ratio associated with cardiotoxic anticancer treatment. The recommended strategy should include a baseline evaluation of cardiac function, followed by regular assessments every 3 months for the first 12 months after therapy, followed by annual assessments. Finally, managing preexisting cardiovascular risk factors is of utmost importance among racial/ethnic minorities, considering the significant role of comorbidities in raising their cardiac toxicity risk. This necessitates the implementation of appropriate strategies for managing these risk factors before, during, and following therapy.

There is an increasing awareness of determinants beyond the realm of the healthcare system, including socioeconomic status, education, geographical segregation, and structural racism. These factors have been demonstrated to impact health status and access to healthcare services. Consequently, it is essential to establish adequately funded research centers to promote multidisciplinary research aimed at comprehending and eradicating racial and ethnic healthcare disparities. It is also imperative that healthcare leaders and medical professionals make concerted efforts to preserve existing coverage options, such as Medicaid, while concurrently working to improve other avenues of coverage for uninsured individuals. In addition, strategies to address language and cultural barriers that may impede the provision of adequate healthcare are warranted. These measures include an increase in the use of medical interpretation services, the diversification of the racial and ethnic composition of the healthcare workforce, and the establishment of training programs and resources for healthcare providers in the area of cross-cultural education.

It is crucial to strengthen patient advocacy and allocate resources for the regular collection and analysis of data on healthcare utilization among various racial and ethnic groups. It is also essential to incorporate data from national surveys, health insurers, and various health settings to obtain a deeper understanding of the issues at hand and the efficacy of proposed solutions. Additional strategies to address the racial/ethnic disparities in cardio-oncology among breast cancer patients encompass several key recommendations. Firstly, it is crucial to enhance awareness through educational initiatives and provide cultural competency training to healthcare professionals. This will enable them to better understand and cater to the specific needs of diverse patient populations. Secondly, efforts should be made to enhance the health literacy of affected groups by expanding educational programming targeted toward these communities. By improving their understanding of cancer care, individuals can make more informed decisions about their treatment options. Thirdly, it is essential to strengthen and sustain healthcare resources in order to enhance the quality of care provided to individuals residing in at-risk areas. This can be achieved through the allocation of adequate funding and resources to healthcare facilities in these regions. Lastly, collaboration with agencies, organizations, coalitions, boards, and councils, such as the American Medical Association (AMA), that are actively engaged in addressing the underlying causes of health disparities is crucial. By working together, these entities can develop comprehensive strategies to mitigate the racial/ethnic disparities observed in cardio-oncology among breast cancer patients.

## Figures and Tables

**Table 1:** Examples of racial disparities in anthracycline-induced cardiotoxicity.

Study	Design	Population	Treatment Dose	Cardiotoxicity Outcomes	Conclusion
Toure et al., 2022 [57]	Retrospective observational study	8604 (274 African American and 8330 White participants)	Doxorubicin (dose ranged from 400 mg/m^2^ to 700 mg/m^2^)	Congestive heart failure (CHF) was detected in 27.8% of White participants and 32.5% of African Americans.	Significant differences between African Americans and White patients in hazard of incident CHF and cumulative incidence of incident CHF.
Collin et al., 2020 [58]	Prospective observational study	8503 (3580 African American and 4923 non-Hispanic White (NHW) participants)	Not specified	Anthracyclines were associated with a greater hazard ratio in African Americans (HR, 1.45) than in White breast cancer survivors (HR, 0.86); data were not statistically significant.	Disparity in chemotherapy-induced cardiotoxicity between African American and White breast cancer survivors contributed to the racial disparity in overall survival between these groups.
Troeschel et al., 2019 [28]	Retrospective observational study	407,587 (36,458 NHW) and 43,562 African American)	Not specified	The 20-year cumulative incidence of CVD-related mortality was 13.3% in African American women and 8.9% in NHW. Higher hazard ratio of cardiovascular mortality in African American breast cancer survivors compared with White breast cancer survivors (HR = 2.73 for those less than 50 and 1.72 for those aged 55 to 68 years).	CVD-related mortality was significantly higher among African American than NHW breast cancer survivors, especially the young.
Berkman et al., 2014 [59]	Retrospective observational study	67,514 (54,518 White and 6113 African American participants)	Not specified	Higher hazard ratio of cardiovascular mortality in African American t cancer survivors compared with White breast cancer survivors (HR = 14.99 for the age group 40–49, HR = 6.43 for the age group 50–59, and HR = 2.26 for the age group 60–69).	Cardiovascular mortality was significantly higher in African American breast cancer survivors, especially among the young.
Braithwaite et al., 2009 [27]	Retrospective observational study	1254 (416 African American and 838 White participants)	Not specified	All-cause mortality was 39.7% in African Americans vs. 33.3% in White patients. The association between hypertension and all-cause survival accounted for 30% of the racial disparity in this outcome.	Hypertension is an independent predictor of the disparity between African American and White breast cancer survivors in cardiovascular outcomes and survival.
Hasan et al., 2004 [26]	Retrospective observational study	499 (100 African Americans and 399 White patients)	The median of cumulative doxorubicin dose infused over 48 h is 374 mg/m^2^; the range is between 264 and 580 mg/m^2^	Higher incidence of doxorubicin-induced cardiotoxicity (LVEF less than 45%) among African American patients (7%) compared to White patients (2.5%).	Doxorubicin-induced cardiotoxicity is more in African American than in White patients.

## Data Availability

Not applicable.
